# A Complexity View into the Physics of the Accelerating Seismic Release Hypothesis: Theoretical Principles

**DOI:** 10.3390/e20100754

**Published:** 2018-10-01

**Authors:** Filippos Vallianatos, Georgios Chatzopoulos

**Affiliations:** UNESCO Chair on Solid Earth Physics and Geohazards Risk Reduction, Technological Educational Institute of Crete, Crete 73100, Greece

**Keywords:** seismicity pattern, accelerating seismicity, Tsallis entropy, non extensive statistical physics

## Abstract

Observational indications support the hypothesis that many large earthquakes are preceded by accelerating-decelerating seismic release rates which are described by a power law time to failure relation. In the present work, a unified theoretical framework is discussed based on the ideas of non-extensive statistical physics along with fundamental principles of physics such as the energy conservation in a faulted crustal volume undergoing stress loading. We define a generalized Benioff strain function $\Omega_{\xi }\left(t\right)=\overset{n\left(t\right)}{\underset{i=1}{\sum }}E_{i}^{\xi }\left(t\right)$, where *E_i_* is the earthquake energy, 0≤ξ≤1. and a time-to-failure power-law of $\Omega_{\xi }\left(t\right)$ derived for a fault system that obeys a hierarchical distribution law extracted from Tsallis entropy. In the time-to-failure power-law followed by $\Omega_{\xi }\left(t\right)$ the existence of a common exponent *m*_ξ_ which is a function of the non-extensive entropic parameter *q* is demonstrated. An analytic expression that connects *m*_ξ_ with the Tsallis entropic parameter *q* and the *b* value of Gutenberg—Richter law is derived. In addition the range of *q* and *b* values that could drive the system into an accelerating stage and to failure is discussed, along with precursory variations of *m*_ξ_ resulting from the precursory *b*-value anomaly. Finally our calculations based on Tsallis entropy and the energy conservation give a new view on the empirical laws derived in the literature, the associated average generalized Benioff strain rate during accelerating period with the background rate and connecting model parameters with the expected magnitude of the main shock.

## 1. Introduction

Earthquake physics is one of the most fascinating fields in Earth Sciences. It’s not only the abruptness of the phenomenon that attracts our interest, but also the devastating consequences that earthquakes can have for the anthropogenic environment. Thus an understanding of its hidden fundamental physics required in order to mitigate the earthquake risk. The earthquake generation process is commonly believed to be a complex phenomenon [1,2,3,4,5,6], although this has been questioned in [7,8,9], manifested in the nonlinear dynamics and in the wide range of spatial and temporal scales that are incorporated in the process [1,2].

The Accelerating Seismic Release (ASR) ideas were applied for first time more than twenty five years ago [3,4,5,6]. The idea of ASR has been adopted and modified properly by many scientists and in different geotectonic environments [3,4,5,6,10,11,12,13,14,15,16,17,18,19,20,21,22,23,24,25] (and references therein). In most of the cases the application of ASR was retrospective, usually after a large earthquake, although there were few attempts at prediction but unfortunately very few of them were successful (see the discussion in [8]), suggesting that further study of the physics of the ASR hypothesis is necessary. A large number of seismological observations show that strong mainshocks are preceded by accelerating generation of intermediate magnitude preshocks [11,15,18] (among many others). Theoretical work suggests that the process of generation of these preshocks can be considered as a critical phenomenon culminating in a mainshock considered as a critical point [26,27,28]. In addition rock mechanics laboratory experiments support the idea that rupture in heterogeneous media is a critical phenomenon [16,29].

Initially the ASR theory has been associated with subcritical crack extension theory where in rocks under constant stress the small cracks expand rapidly before the occurrence of the main fracture [5,6]. This approach was associated with the critical point concept, since the preparation of an earthquake was described as a critical phenomenon that leads to a critical point which is the main earthquake that occurs when fracturing becomes coherently self-organized at different scales [26,30]. The latter has been correlated with power-law increase in the cumulative Benioff strain release rate prior to the characteristic earthquake [6,25]. Furthermore, the ASR hypothesis could be explained by the phase transitions theory and spinodals lines where fault failures are produced from continue evolving and correlated system [22] (and references therein). We note that in [7,8,9] an alternative view is proposed, called non-critical precursory accelerating seismicity theory (NC-PAST). It is based on the assumption that foreshocks are due to the cumulated effects of constant loading on the fault zone that hosts the mainshock. In this view, foreshocks are the passive tracers of the preparatory process of the mainshock and therefore carry information on the upcoming event. In the NC-PAST view some large earthquakes are potentially predictable, even if in practice no probabilistic model is yet available. We point out that even NC-PAST presents an alternative view to criticality, the concept of critical point could be used to explain the origin of ASR [8] and it is a reasonable theory to explain the behavior of earthquake populations. An extended discussion on this debate, between the critical point holistic view, where the seismic patterns (universal power-laws) are the signatures of physical interactions at all scales in the lithosphere, and the NC-PAST reductionalist view, where different loading processes are superimposed depending on the geometry of the fault network, and where patterns are progressively defined through space and time by the sum of the different loading components of the system is presented in [8] along with a long list of references on ASR. We clarify that the present work is based on the critical earthquake model view [1,2,3,4,5,6,10,11,12,13,14,15,16,17,18,19,20,21] which has recently supported in terms of natural time analysis [31] and we suggest to the reader the works [7,8,9] for an alternative approach.

The critical earthquake model is based on principles of statistical physics [26,27,28] and has been proposed to explain accelerating intermediate magnitude seismicity observed before strong mainshocks [11,17,21] (among others). Such behavior has been also supported by independent observations, which suggest that rupture in heterogeneous media is a critical phenomenon [30]. Thus, the critical earthquake model is supported by seismological observations, by principles of statistical physics and rock mechanics experiments.

Several researchers have investigated properties of the model [6,10,12,25]. Based on a damage mechanics model, in [6] proposed a power law for the time variation of the cumulative Benioff strain, which defined as the cumulative of the square root of energy, released by preshocks in the critical region. The expression proposed was:

(1) $$\Omega \left(t\right)=\Omega_{f}-B{\left(t_{f}-t\right)}^{m}$$

where *t_f_* is the failure time (occurrence time of the mainshock) and $\Omega_{f}$, *B*, and *m* are model parameters. The exponent *m* takes values much smaller than 1 for accelerated energy release, whereas values at about *m*~1 correspond to a steady (normal) time variation of the Benioff strain (seismic energy) release. For *m* > 1 a deceleration of seismicity is defined. However, results from numerous experimental studies show that approaching failure *m* has a mean value close to 0.30, in agreement with several theoretical considerations and laboratory results [10,32].

It is obvious that the study of seismicity patterns requires methods of statistical physics and seismicity processes can be seen as the outcome of the irreversible dynamics of a long-range, interacting, disordered system [33]. The main motivation of this work is starting from fundamental ideas that combine a theoretical frame for the ASR method with complexity theory, introducing concepts such as that of non-extensive statistical mechanics. Our theoretical findings are discussed in comparison with previously observed empirical scaling expressions.

Non-extensive statistical physics (NESP) is the appropriate methodology to describe seismicity patterns, where long-range dependence effects are important. NESP was originally introduced in [34], recently summarized in [35], while its validity in Earth Sciences is reviewed in [36,37]. It is based on Tsallis entropy, a generalization of the classic Boltzmann-Gibbs entropy and has the main advantage that it considers all-length scale correlations among the elements of a system, leading to an asymptotic power-law behavior, very common in Earth Sciences. Non-extensivity represents one of the most intriguing characteristics of systems that have experienced long-range temporal correlations [38] which is observed in seismicity and recently has been verified in terms of natural time analysis [39]. The applicability of NESP in Earth physics has been demonstrated in a series of recent publications on seismicity [40,41,42,43,44,45,46,47], natural hazards [48,49], plate tectonics [50], geomagnetic reversals [51], rock physics [52,53], applied geophysics [54] and fault-length distributions [55,56,57].

The question whether accelerating/decelerating seismicity could be described in terms of NESP presents a challenge. This is the problem addressed here. Taking into account the different complex models proposed [10,11,12,13,14,15,16,17,18,19,20,21,22,23,24,25,26,27,28,29,30], reviewed and criticized in [7,8,9] it is very attractive to focus our interests on the results obtained introducing ideas of complexity and NESP. For this scope we generalize a model originally proposed in [16] where fundamental concepts as that of energy conservation are used to understand the accelerating seismicity and to demonstrate the physics that governs it.

It is primary target of the present work to discuss a unified theoretical framework based on the ideas of NESP and Tsallis entropy, along with fundamental principles of physics such as the energy conservation in a faulted crustal volume undergoing tectonic stress loading, in order to derive the time-to-failure power-law of a generalized Benioff strain expression $\Omega_{\xi }\left(t\right)$ in a fault system with earthquake volumes that obey a hierarchical distribution law. We note that the present analysis is based on already existing observations regarding ASR. Herein we will extent the model proposed in [16] in order to include the generalized Benioff strain $\Omega_{\xi }\left(t\right)$ and to study its fundamental physical properties, in view of the NESP approach. Considering the analytic conditions near the time of failure, we derive from first principles the generalized time-to-failure power-law and we present that a common critical exponent *m_ξ_* exists, which is a function of the non-extensive entropic parameter *q* or in an equivalent way of the *b*-value that appears in the Gutenberg-Richter law. Our results based on Tsallis entropy and the energy conservation, present a physical reason for the validity of the empirical laws observed in a number of previous works [11,15,17,21] that connect the empirical parameters of the time-to-failure power-law expression with the magnitude of the main shock.

## 2. A Non Extensive Statistical Physics Formulation of Seismicity Temporal Pattern

This section is organized as follows: in Section 2.1 we define the generalized Benioff deformation $\Omega_{\xi }\left(t\right)$ and its basic physical properties based on the fundamental principle of energy conservation in a stressed fractured volume. In the following Section 2.2 and Section 2.3 the non-extensive statistical physics introduced and linked to the generalized Benioff deformation leading to a relation between the power law exponent and the Tsallis entropic parameter *q* or equivalently the *b*-value in the Gutenberg—Richter law. Finally in Section 2.4 the fundamental properties of $\Omega_{\xi }\left(t\right)$ are presented and discussed in view of already published empirical laws.

### 2.1. The Generalized Benioff Deformation

The earthquake preparatory process results in the generation of several preshock seismicity patterns. One of these patterns is characterized by accelerating seismicity expressed by the generation of moderate magnitude earthquakes that occur before a mainshock in a critically deformed region [12]. In contrast a second pattern concerns the seismic quiescence, expressed by the decrease of the observed seismicity [58,59,60,61,62]. It has been suggested [10,11,12,13,14,15,16,17,18,19,20,21,22,23,24,25,26,27,28,58,61] that strong mainshocks are preceded by a seismicity pattern where accelerating strain in the region is accompanied by decelerating strain in the narrow part (in the vicinity of epicenter) seismogenic region. It is obvious that the use of the term accelerating–decelerating seismic crustal deformation reflects the physical process that takes place at the critical preshock area.

To describe the accelerating–decelerating seismic crustal deformation the following equation for modeling the process of energy release during the large earthquake preparation (the time to failure model) has been used [10,11,12,13,14,15,16,17,18,19,20,21,22,23,24,25,26,27,28]: $\frac{d\Omega }{dt}=\frac{k}{{\left(t_{f}-t\right)}^{1-m}}$ or the integral form *Ω*$\left(t\right)=\Omega_{f}-B{\left(t_{f}-t\right)}^{m}$ (as presented in Equation (1)), where $B=\frac{k}{m}$.

The cumulative Benioff strain, Ω(t), is a measure of the preshock seismicity at time t, defined as: $\Omega \left(t\right)=\overset{n\left(t\right)}{\underset{i=1}{\sum }}E_{i}^{1/2}\left(t\right)$, $E_{i}$ is the seismic energy of the *i-th* preshock and n(t) is the number of events till time t. The parameter $t_{f}$ is the occurrence time of the mainshock and B and *m* are parameters which can be calculated from available observations. For 0 < *m* < 1 an accelerated seismicity pattern is observed, while for *m* > 1 a decelerated pattern appears.

Seismic energy is usually calculated from the corresponding magnitude of the earthquakes. Instead of the Benioff strain (roughly proportional to *E*^½^), other measures, such as the seismic moment (~*E*^1^) or the number of events (~*E*^0^), have been used to describe accelerating–decelerating seismicity patterns. The Ω(t) could be generalized, introducing a new quantity $\Omega_{\xi }\left(t\right)=\overset{n\left(t\right)}{\underset{i=1}{\sum }}E_{i}^{\xi }\left(t\right)$ where 0≤ξ≤1 that we call generalized Benioff deformation. When ξ=0, then $\Omega_{0}\left(t\right)=N(<t)$ where N(<t) is the cumulative number of earthquakes till time t, for ξ=1/2 we have $\Omega_{1/2}\left(t\right)=\Omega \left(t\right)$ i.e., the well-known cumulative Benioff strain and when $\xi =1,\;\Omega_{1}\left(t\right)=\sum E\left(t\right)$ represents the cumulative energy released.

Since the system of faults has a fractal structure, [63] and in the fault zone, at a first approximation, a hierarchical scaling of fractures takes place, it has been suggested that the process of the main shock preparation is a critical phenomenon [28], which occurs when fracturing becomes coherently self-organized at different scales. This process develops from below upwards’ following the energy scales of self-organized fractures and is eventually concentrated in the vicinity of the hypocenter of the main shock. Seismicity patterns associated with the nucleation of strong earthquakes are often recorded over the earthquake epicenter in a fairly large area *V*. We note that the earthquake epicenter can lie in both central and peripheral parts of this area. The size of the volume *V* is an order of magnitude greater than that of the earthquake source region. The stressed crustal volume *V* is the region where the preparation process of large earthquake occurs. However, in addition to the volume *V*, the earthquake nucleation process should give rise to a potential earthquake source region *V_eff_* developing with time *t* in which the macrofractures are nucleated. The maximum diameter L of *V_eff_* is of the same order as that of the earthquake source.

**Figure 1:**
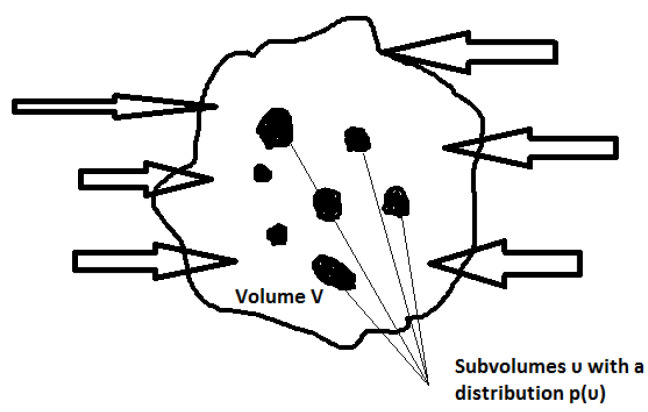
The tectonically stressed volume *V*. Within *V* seismic energy is released in the hierarchically distributed subvolumes *υ* that form the potential earthquake source region *V_eff_* (see text).

In the first initial phase the temporal and spatial distribution of seismic activity within *V* is approximately uniform. During the initial phase the flow of tectonic elastic energy into *V* is released with weak earthquakes and possibly with an additional aseismic deformation (e.g., creep). It is straightforward to accept that due to the inhomogeneity of the crust the elastic energy is concentrated in some subvolumes υ within *V* (see Figure 1). The latter leads to the increase of stress in subvolumes and at a critical time the configuration of the stress field specifies the parameters of the future main shock. To express the energy which supports stress we define as $U_{s}^{in}$ the elastic energy surface density which tectonically flows within *V* as $U_{s}^{in}=\frac{dU^{in}}{dA}$ where *A* is the area associated with the external bound of the volume *V*. Τhe energy is released as seismic activity and we define $U_{v}^{out}$ as the volume density of the elastic energy seismically released as a result of the earthquake activity within $V_{eff}$. We note that $V_{eff}$ is formed by the set of all the earthquake subvolumes υ within *V*, $(V_{eff}\mathbb{C}\;V$), while an aseismic term R(t) exists to describe the part of the inflow energy to *V* which is not related to the earthquake activity.

According to the fundamental principle of energy conservation we have:

(2) $$U_{s}^{in}A\left(V\right)=U_{V}^{out}V_{eff}+R\left(t\right)$$

or:

(2a) $$U_{s}^{in}A\left(V\right)=U_{V}^{out}V_{eff}+\lambda V$$

where R(t)=λ V assumed. Motivated from the Voight relation [64], we generalized in Equation (3) the assumption suggested in [16] (for *ξ* = 1/2), accepting its validity for the generalized Benioff strain $\Omega_{\xi }\left(t\right):$

(3) $$\frac{d\Omega_{\xi }\left(t\right)}{dt}=\gamma {\left[U_{V}^{out}\left(t\right)\right]}^{\alpha }=\gamma_{1}{\left[\frac{U_{s}^{in}A\left(V\right)-\lambda \;V}{V_{eff}}\right]}^{\alpha }$$

Equation (3) relates the rate of the generalized Benioff deformation with the volume density of the elastic energy released and it is similar with that used in damage mechanics, where the evolution of the damage variable is related with the square of the strain [65,66,67]. If *L* is the characteristic size of the volume *V* then $A\left(V\right)\sim L^{d_{e}-1}$ and *V*$\sim L^{d_{e}}$ where *d_e_* is the Euclidean dimension of *V* which is *d_e_* = 3 when the earthquake activity in embedded in a 3 dimensional space and *d_e_* = 2 when it is located in an almost 2-dimensional surface. We clarify that hereafter the term “volume” has to be viewed as the geometrical size related with the spatial distribution of earthquake events and as mentioned it is the geometrical volume in the case of a 3-dimensional distribution of preshocks.

### 2.2. A Non Extensive Statistical Physics View of Generalized Benioff Deformation

We proceed now to the estimation of the probability distribution *p(υ)* of the sub-volumes *υ* that form the *V_eff_*. To this direction, we introduce the principles of non-extensive statistical mechanics in our analysis. Its cornerstone which is recapitulated here, is the non-additive entropy *S_q_* [34,35], which is non-additive in the sense that it is not proportional to the number of the system’s elements, as in the Boltzmann-Gibbs entropy *S_BG_*. The Tsallis entropy *S_q_* reads as:

$$S_{q}=k_{B}\frac{1-\sum_{i=1}^{W}p_{i}^{q}}{q-1},\;q\in R$$

or in equivalent form as $S_{q}=-k_{B}\int p^{q}ln_{q}p\;dx$ for a continuum variable *x*, with $ln_{q}X=\frac{X^{1-q}-1}{1-q}$ the definition of the q-logarithmic function and *k_B_* is Boltzmann’s constant; *p_i_* and *p(x*) are the probabilities of *x*; *W* is the total number of microscopic configurations; and *q* the entropic index. This last index is a measure of the non-additivity of the system and for the particular case *q* = 1, the Boltzmann-Gibbs entropy *S_BG_* is obtained; $S_{BG}=-k_{B}\overset{W}{\underset{i=1}{\sum }}p_{i}lnp_{i}$. We note that for *q* = 1, we obtain the well-known exponential distribution [34]. The cases *q* > 1 and *q* < 1 correspond to sub-additivity and super-additivity, respectively. Although Tsallis entropy shares a lot of common properties with the Boltzmann-Gibbs entropy, *S_BG_* is additive, whereas *S_q_* (*q* ≠ 1) is non-additive [34]. According to this property, *S_BG_* exhibits only short-range correlations, and the total entropy depends on the size of the systems’ elements. Alternatively, *S_q_* allows all-length scale correlations and seems more adequate for complex dynamical systems, especially when long-range correlations between the elements of the system are present.

For a system composed of two statistically independent subsystems, *Σ*_1_ and *Σ*_2_, the Tsallis entropy satisfies the equation [34]:

$$S_{q}(\Sigma_{1}+\Sigma_{2})=S_{q}\left(\Sigma_{1}\right)+S_{q}\left(\Sigma_{2}\right)+\frac{1-q}{k_{B}}S_{q}\left(\Sigma_{1}\right)S_{q}\left(\Sigma_{2}\right)$$

The non-additivity is indicated by the last term on the right side of the equation above and represents the interaction between the two subsystems $\Sigma_{1}$ and $\Sigma_{2}$. In order to estimate the probability distribution *p*(υ) of the seismic subvolume *υ*, we maximized the non-extensive entropy under the appropriate constraints, using the Lagrange-multipliers method with the Lagrangian [34,35]:

$$L_{q}=-\int_{0}^{\infty }p^{q}\left(\upsilon \right)ln_{q}p\left(\upsilon \right)d\upsilon -\lambda_{ο}\;(\int_{0}^{\infty }p\left(\upsilon \right)d\upsilon -1)-\lambda_{1}\;(\int_{0}^{\infty }\upsilon P_{q}\left(\upsilon \right)d\upsilon -{\langle \upsilon \rangle }_{q})$$

The first constraint used refers to the normalization condition that reads as: $\int_{0}^{\infty }p\left(\upsilon \right)d\upsilon =1$. Introducing the generalized expectation value (*q*-expectation value), *υ_q_* which is defined as: ${\langle \upsilon \rangle }_{q}=\upsilon_{q}=\int_{0}^{\infty }\upsilon P_{q}\left(\upsilon \right)d\upsilon$, where the escort probability is given in [35] as: $P_{q}\left(\upsilon \right)=\frac{p^{q}\left(\upsilon \right)}{\int_{0}^{\infty }p^{q}\;\left(\upsilon \right)d\upsilon }$, the extremization of *S_q_* with the above constraints yields to the probability distribution of *p*(*υ*) as [68,69]:

(4) $$p\left(\upsilon \right)=C_{q}{\left[1-\frac{1-q}{2-q}\left(\frac{\upsilon }{\upsilon_{q}}\right)\right]}^{\frac{1}{1-q}}$$

where $C_{q}$ is a normalization coefficient. We recall that the *Q*-exponential function is defined as:

$$exp_{Q}\left(X\right)=\{\begin{array}{l} \left[1+\left(1-Q\right)X\right]1/(1-Q)\;\mathrm{if}\;(1+(1-Q)\;X\geq 0)\\ \;0\;\mathrm{if}\;(1+\left(1-Q\right)\;X<0) \end{array}$$

The normalized cumulative number of seismic subvolumes *υ* can be obtained by integrating the probability density function *p*(υ) as:

$$P\left(>\upsilon \right)=\frac{N\left(>\upsilon \right)}{N_{0}}={\left[1+\left(\frac{q-1}{2-q}\right){\left(\frac{\upsilon }{\upsilon_{q}}\right)}^{}\right]}^{\frac{q-2}{q-1}}$$

where *N*(>*υ*) is the number of events with seismic volume larger than *υ*. In the latter expression, if we define $=2-\frac{1}{Q}$, this leads to:

$$P\left(>\upsilon \right)=exp_{Q}\left(-{\left(\frac{\upsilon }{\upsilon_{q}}\right)}^{}\right)={\left[1+\left(Q-1\right){\left(\frac{\upsilon }{\upsilon_{q}}\right)}^{}\right]}^{-\frac{1}{Q-1}}$$

having a typical *Q*-exponential form.

In the frame of non extensive statistical mechanics approach for earthquake volumes bigger than a given one *υ_o_* we find a power law description of the distribution function and in such a case the cumulative distribution is $P\left(>\upsilon \right)≅C{\left(\frac{\upsilon }{\upsilon_{q}}\right)}^{-\frac{2-q}{q-1}}\sim \upsilon^{-\beta }$ with an exponent $\beta =\frac{2-q}{q-1}$. To have an estimate of *υ_ο_* we select the volume where the power law approximation of *P(>υ)* takes the value *P(>υ)* = 1 leading to υο=υq(2−qq−1)32. We observe that *β* ≤ 1 leading to $\frac{3}{2}\leq q$, in agreement with previous published results on earth physics processes at a broad range of scales from laboratory up to geodynamic one [70,71]. It is obvious that the volume distribution *p(υ)* could lead to an estimation of *V_eff_* as $V_{eff}=\int_{V_{min}}^{V}\upsilon p\left(\upsilon \right)d\upsilon$ which for large volumes (i.e., moderate to significant events) has an asymptotic behavior Veff~V(2q−3)(q−1). or *V_eff_*~$L^{d}$ where $d=d_{e}\frac{2q-3}{q-1}$ which generalizes and justify the expression introduced in [16]. The latter expression implies that when *d* > 0 (*q* > 3/2), $V_{eff}\sim L^{d}$ presents a fractal distribution of earthquake volumes with a fractal dimension $d_{e}-1<d<d_{e}$, leading to $\frac{2d_{e}+1}{d_{e}+1}<q<2$. The latter expression suggests that within NESP approach the entropic parameter *q* is bounded by the Euclidean dimension $d_{e}$ of the dmed system. When $d_{e}=3$ then $\frac{7}{4}<q<2$, while for $d_{e}=2$ we constrain *q* in the range $\frac{5}{3}<q<2$.

After introducing Equation (2a) into Equation (3), the generalized Benioff stress rate could be expressed as follows:

(5) $$\frac{d\Omega_{\xi }\left(t\right)}{dt}=\gamma {\left[U_{s}^{in}\frac{1}{L^{d-d_{e}+1}}-\lambda L^{d_{e}-d}\right]}^{\alpha }$$

When the time *t* approaches the time to failure $t_{f}$ and since $U_{s}^{in}\left(t=t_{f}\right)\neq 0$ following [16] an expansion of $U_{s}^{in}$(*t*) when $t\to t_{f}$ is:

(6) $$U_{s}^{in}=U_{0in}+U_{1in}\left(\frac{t_{f}-t}{T_{c}}\right)+O\left({\left(\frac{t_{f}-t}{T_{c}}\right)}^{n}\right)$$

**Figure 2:**
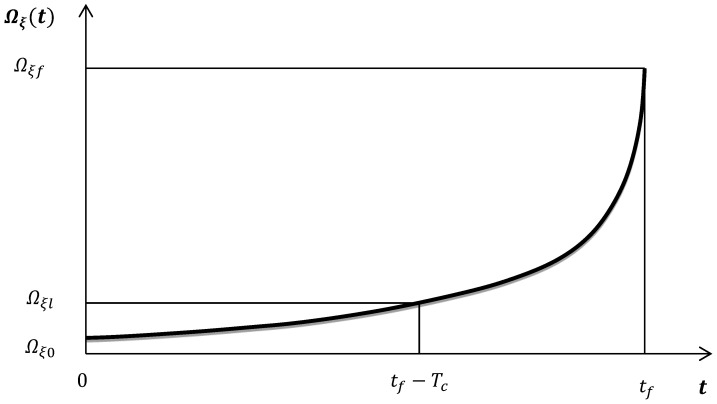
Evolution of the generalized Benioff strain $\Omega_{\xi }\left(t\right)$. The initial part is linear and the deviation from linearity starts at *t* = *t_f_* − *T_c_* defining the start of the accelerating deformation stage, where $T_{c}$ is the characteristic time expressing the duration of the main shock preparation process.

From Equation (6) it is obvious that $U_{0in}=U_{s}\left(t=t_{f}\right)$, expressing the elastic energy of tectonic origin inserted into the deformed area at the time of failure. The third term O(x). presents all the highest order terms in the expansion that are very small and could be omitted, while the parameter $T_{c}$ is the characteristic time that defines the duration of the main shock preparation process starting from the time where deviation of $\Omega_{\xi }\left(t\right)$ from linearity appears (see Figure 2).

Approaching the main shock the volume where energy is released, defines a singular point [16], and the analyticity assumption of L(t) as $t\to t_{f}$ leads to:

(7) $$L\left(t\right)=L_{0}\left(\frac{t_{f}-t}{T_{c}}\right)+O\left({\left(\frac{t_{f}-t}{T_{c}}\right)}^{n}\right)$$

A detailed discussion of Equation (7) is given in [72,73] where a scale invariance of the process is assumed in analogy with the theory of phase transitions. We note that when $t\to t_{f}$, L(t)→0 and $L\left(t=t_{f}-T_{c}\right)=L_{0}$. Substituting Equations (6) and (7) into (5) we obtain:

(8) $$\frac{d\Omega_{\xi }\left(t\right)}{dt}=\gamma {\left\{\frac{U_{0in}}{{\left[L_{0}\left(\frac{t_{f}-t}{T_{c}}\right)\right]}^{d-d_{e}+1}}+\frac{U_{1in}\left(\frac{t_{f}-t}{T_{c}}\right)}{{\left[L_{0}\left(\frac{t_{f}-t}{T_{c}}\right)\right]}^{d-d_{e}+1}}-\lambda \;{\left[L_{0}\left(\frac{t_{f}-t}{T_{c}}\right)\right]}^{d_{e}-d}\right\}}^{a}$$

As $t\to t_{f}$ then $\left(\frac{t_{f}-t}{T_{c}}\right)\to 0$. Taking into account that $d_{e}-d$ > 0 and $d+1>d_{e}$ the first term in (8) dominates and the integration leads to:

(9) $$\Omega_{\xi }\left(t\right)=\Omega_{\xi }\left(t=t_{f}\right)-\gamma \frac{T_{c}{\left(U_{0in}\right)}^{a}}{L_{0}^{a\left(d-d_{e}+1\right)}}\frac{1}{a\left(d_{e}-1-d\right)+1}{\left(\frac{t_{f}-t}{T_{c}}\right)}^{1+a\left(d_{e}-1-d\right)}$$

which has the classical form proposed in [4,5,6,14] where:

(10a) $$\Omega_{\xi }\left(t\right)=\Omega_{\xi f}-B{\left(t_{f}-t\right)}^{m_{\xi }}\;\mathrm{with}\;\Omega_{\xi f}=\Omega_{\xi }\left(t=t_{f}\right)$$

(10b) $$B=\gamma \frac{T_{c}^{a\left(d-d_{e}+1\right)}{\left(U_{0in}\right)}^{a}}{L_{0}^{a\left(d-d_{e}+1\right)}}\frac{1}{a\left(d_{e}-1-d\right)+1}$$

(10c) $$m_{\xi }=a\left(d_{e}-1-d\right)+1$$

The expression (10c) suggests that, $m_{\xi }$ is independent of *ξ* (0 ≤ *ξ* ≤ 1) introduced in the definition of the generalized Benioff strain $\Omega_{\xi }\left(t\right)$ but controlled by the Euclidean dimension $d_{e}$. of the deformed system and the entropic parameter *q* which as a measure of long range interactions and of the complexity of the system, controls the distribution of seismic subvolumes *υ* and their fractality. It is worth to mention that the shape of the acceleration curve is controlled primarily by the exponent $m_{\xi }$. Therefore, two differently sized main shocks with the same $m_{\xi }$ value will have similarly shaped acceleration curves but with different scale.

### 2.3. A NESP View of the ASR Parameters

The NESP approach could also be used to formulate the earthquake frequency-magnitude distribution [44]. Moreover, [44] introduced an energy distribution function that shows the influence of the size distribution of fragments on the energy distribution of earthquakes, including the Gutenberg—Richter (GR) law as a particular case. [45] revised the fragment-asperity model using a more realistic relationship between earthquake energy (*E*) and fragment size. Many recent works indicated that the *q* parameter can be used as a measure of the stability of an active tectonic area [40,41,44,45,70,74]. A significant increase of *q* indicates strong interactions between the fault blocks (earthquake volumes) and implies a transition away from equilibrium [36,37,70]. Here we will modify the above mentioned models in order to formulate a frequency-magnitude distribution, taking into account the earthquake volume distribution *p(υ)* and introducing a scaling law between the released relative energy (*E*) and the earthquake volume (*υ*) as has been proposed in [45] in agreement with the scaling relationship between seismic moment and rupture length. From Equation (4) the energy distribution function of the earthquakes can be written as follows:

(11) $$p\left(E\right)=C_{q}{\left[1-\frac{1-q}{2-q}\left(\frac{E}{E_{q}}\right)\right]}^{\frac{1}{1-q}}$$

Since the probability of the energy is $p\left(E\right)=n\left(E\right)/N_{o}$, where n(E) corresponds to the number of earthquakes with energy E and $N_{o}$ is the total number of earthquakes, the normalized cumulative number of earthquakes is given as:

(12) $$\frac{N\left(>E\right)}{N_{o}}=\overset{\infty }{\underset{E}{\int }}p\left(\varepsilon \right)d\varepsilon$$

where N(>E) is the number of earthquakes with energy greater than *E*. Combining Equations (11) and (12) the following expression for the earthquake frequency-energy distribution *is* derived:

(13) $$\frac{N(>E)}{N_{o}}={\left[1-\frac{\left(1-q\right)}{\left(2-q\right)}{\left(\frac{E}{E_{q}}\right)}^{}\right]}^{\frac{2-q}{1-q}}.$$

which for $\frac{\left(q-1\right)}{\left(2-q\right)}\left(\frac{E}{E_{q}}\right)\gg 1$ suggests a scaling law $N(>E)\sim E^{-\frac{2-q}{q-1}}$ in agreement with the well known power law scaling $N(>E)\sim E^{-\beta }$ with $\beta =\frac{2-q}{q-1}$ [36,37,70,75]. As proposed in [76] the earthquake magnitude *M* and the released seismic energy *E*, are related as $M\sim \frac{2}{3}\log\left(E\right)$, leading to a *b*-value in the Gutenberg—Richter law:

(14) $$b=\frac{3}{2}\beta =\frac{3}{2}\frac{2-q}{q-1}$$

**Figure 3:**
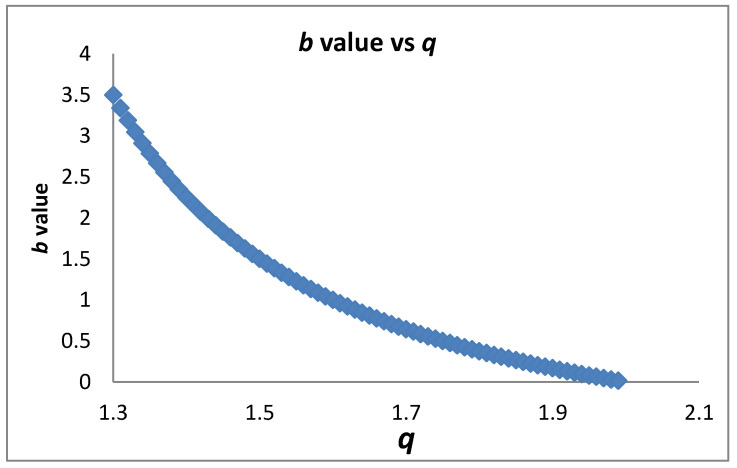
The *b* value vs *q* as defined in Equation (14) (see text).

Figure 3 presents the dependence of the *b* value on *q* as given in (14). Substituting $d=d_{e}\frac{2q-3}{q-1}$ into $m_{\xi }$ as given by Equation (10c) and taking into account (14) we find:

(15) $$m_{\xi }=1-\alpha +\alpha d_{e}\frac{2-q}{q-1}=1+\alpha d_{e}\left(\beta -\frac{1}{d_{e}}\right)=1+\alpha \;\left(\frac{2}{3}b\;d_{e}-1\right)$$

which connects the non-extensive parameter *q* and the *b*-value of the Gutemberg—Richter law with the $m_{\xi }$ parameter of the generalized Benioff strain. We note that in [6] using synthetic data a relationship between *b*-value and $m_{1/2}$ was claimed. For the parameter $m_{\xi }$ a positive definition is required ($m_{\xi }>0)$ and thus $d_{e}-1<d<d_{e}-1-\frac{1}{a}$. Theoretical results and experimental observation [3,4,5,6,10,11,12,13,14,15,16,17,18,19,20,21,22,23,24,25] suggest that $m_{1/2}=0.25-0.30$ and d=2.3−2.4, while $m_{\xi }$ should be the same for different ξ values, in agreement with observations that indicate $m_{0}=0.30$ [75]. Substituting $m_{\xi }\approx 0.3\;\mathrm{and}\;d\approx 2.3\;\mathrm{for}\;d_{e}=3$ or $d\approx 1.3\;\mathrm{for}\;d_{e}=2$ to (10c) we find a=2.0−2.1. From here on we will keep a constant value a≈2.0 in agreement with damage mechanics models where the evolution of the damage variable is related with the square of the strain [65,66,67].

**Figure 4:**
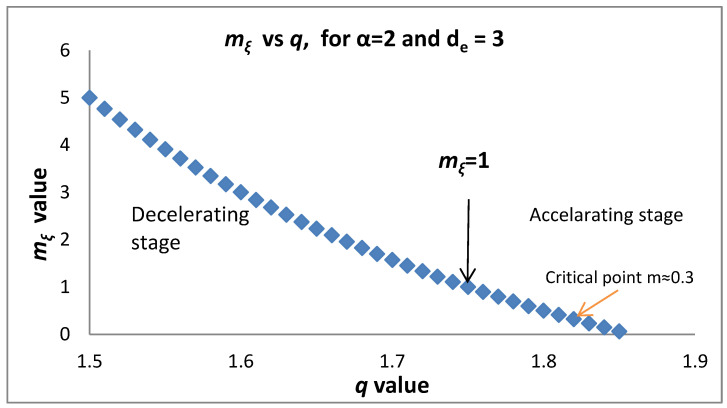
Dependence of *m_ξ_* on the entropic parameter *q* along with the accelerating/decelerating stages (see text).

**Figure 5:**
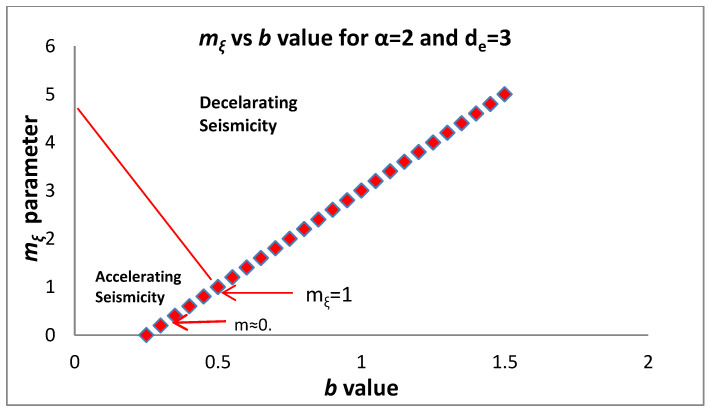
Dependence of *m_ξ_* on the *b* value along with the accelerating/decelerating ranges of the earthquake system.

Equation (15) along with the constrain $m_{\xi }>0$ leads to a lower bound for the *b* value and an upper bound for the *q* value, respectively, given as:

(16) $$b>\frac{3\left(a-1\right)}{2ad_{e}}\;\mathrm{and}\;q<\frac{2ad_{e}+a-1}{ad_{e}+a-1}$$

which for α = 2 and $d_{e}=3\;\mathrm{gives}\;b>0.25$ while for $d_{e}=2\;,\;b>0.375.$ The maximum permitted value of *q* is *q*_max_= 13/7 for $d_{e}=3\;\mathrm{and}$ *q*_max_ = 9/5 for $d_{e}=2$. For accelerating (decelerating) seismicity $m_{\xi }<1$ ($m_{\xi }>1$). Applying Equation (15) shows that in accelerating seismicity $q>\frac{2d_{e}+1}{d_{e}+1}$ which implies a lower bound of the observed q introduced by the topological Euclidean dimension $d_{e}$ of the space where the earthquakes are embedded. For $d_{e}=3,\;q>\frac{7}{4}$ while for $d_{e}=2,\;q>\frac{5}{3}$. In a similar way we have $q<\frac{2d_{e}+1}{d_{e}+1}$ for decelerating seismicity. Furthermore, Equation (15) for $m_{\xi }<1$ (accelerating seismicity) leads to $b<\frac{3}{2d_{e}}$ which (as we approach failure) for $d_{e}=3$ leads to b<0.5 and for $d_{e}=2\;\mathrm{to}\;b<0.75$. The above expressions introduce a critical value for *q* and for *b* where a transition from decelerating to accelerating seismicity occurs. It is obvious that the decelerating seismicity which is described by $m_{\xi }>1$ for $d_{e}=3$ leads to b>0.5 and for $d_{e}=2\;\mathrm{to}\;b>0.75$. Figure 4 and Figure 5 present the dependence of *m_ξ_* on *q* and *b*, respectively.

**Figure 6:**
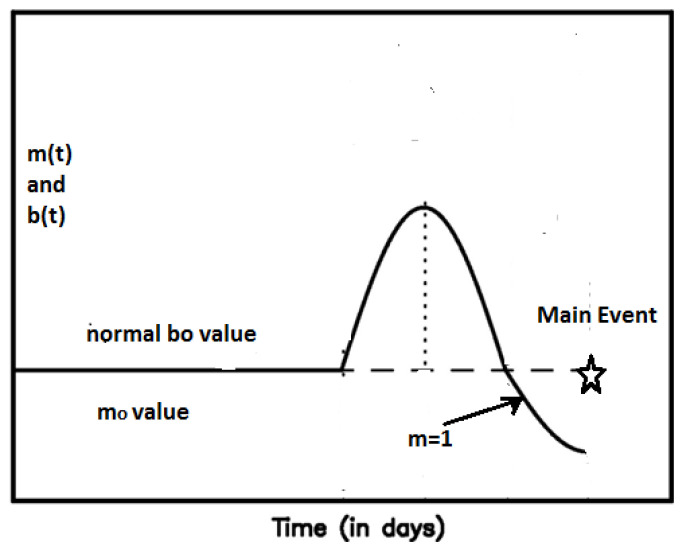
Pattern of the variation of *b* and *m_ξ_* values with time following the mechanism for *b* value preseismic changes proposed in [80] (modified from [80]).

Furthermore the above analysis could be applied to connect changes of *m_ξ_* to *b*-value variations which have been reported as precursory effects in a number of significant earthquake events [76,77,78,79]. Equation (15) suggests that variations of the *b* value are associated with the temporal evolution of *m_ξ_* during the main event preparation period *T_c_*, following the *b* values changes as suggested in [80]. We write *b(t)* = *b_o_* + *Λ(t)* where *b_o_* represents the background *b*-value and *Λ(t)* reflects the time dependent part of *b*-value that varies during the preparation of the main earthquake event (see Figure 6). Substituting in (15) we find:

$$m_{\xi }=m_{\xi o}+\frac{2a}{3}d_{e}\Lambda \left(t\right)\;\mathrm{where}\;m_{\xi o}=1+\alpha \left(\frac{2}{3}b_{o}d_{e}-1\right).$$

For α = 2, *b_o_* = 1 and $d_{e}=3\;\mathrm{we}\;\mathrm{find}\;m_{\xi o}=3$ while for $d_{e}=2$, $m_{\xi o}=1.67$, both describing a decelerating stage of seismicity. As we approach the failure time *t_f_,* observational results suggest that $m_{\xi }\approx 0.30-0.35$, leading to $\Lambda \left(t_{f}\right)\approx -\frac{1}{6}$ for $d_{e}=3$ and $\Lambda \left(t_{f}\right)\approx -1/4$ for $d_{e}=2$, respectively. Figure 6 exhibits the general pattern of the temporal variation of the *m_ξ_* parameter following the temporal variation of the *b*-value as suggested in [80]. Most of the time after the last main event the *m_ξ_* value varies around *m_ξo_*, which corresponds to the average *b_o_* value measured over a long time period. As the *b* value increases from *b_o_* to a maximum value (thus a seismic quiescense appears in agreement with [24]), the parameter *m_ξ_(t)* increases too in a way following Equation (15). After passing a maximum value *m_ξmax_*, a decreasing phase of both *b* and *m_ξ_* starts, crossing the value *m_ξο_* and approaching the transition time *t_c_* where *m_ξ_(t_c_)* = 1, which defines the passing from the decelerating to an accelerating stage. At the next step *m_ξ_(t)* is approaching the value $m_{\xi }\left(t\to t_{f}\right)$ which lies in the range of 0.25–0.35 and suggests the approach to a final stage of the preparation of the mainshock. The latter is in agreement with [81,82] where *b* values based on seismicity over a period from 2006 till immediately before the Tohoku earthquake, revealed a zone of low *b* value (*b* ≈ 0.5–0.6) in and around the focal area as an indicator of highly stressed patches in the zone, in remarkable similarity to *b* values obtained in laboratory experiments [83]. We note that for *b* ≈ 0.5 Equation (15) gives *m_1/2_* ≈ 0.3 in agreement with the value *m* = 0.24 ± 0.09 given in [84].

### 2.4. Fundamental Properties of the $\Omega_{\xi }$ Function

Here we study some fundamental properties of the $\Omega_{\xi }\left(t\right)$ function that could be used to understand the physics of many empirical laws presented in [11,15,17,19,21]. From Equation (10b) we find:

(17) $$\log B=\left[\log\gamma \frac{T_{c}^{1-m_{\xi }}{\left(U_{0in}\right)}^{a}}{m_{\xi }\;L_{0}^{1-m_{\xi }}}\right]=\log\left(\frac{\gamma }{m_{\xi }}\right)+\alpha \log(U_{0in})+\left(1-m_{\xi }\right)\log T_{c}-\left(1-m_{\xi }\right)\log L_{0}$$

The energy of the main shock is $E_{m}\sim U_{0in}\;L_{0}{}^{2}$ or $logE_{m}\sim \left(\log U_{0in}+2\log L_{0}\right)$. Experimental results and theoretical estimations suggest that the preparation time has a very weak dependence of the magnitude of the main shock. Substituting to Equation (17) the scaling laws $\log E_{m}≅1.5M+const,\;\mathrm{and}\;\log L_{0}\approx 0.5\;M+const$, we find $\log B\approx \left(\frac{a+m_{\xi }-1}{2}\right)M+const$. Since $a\approx 2,\;m_{\xi }=0.25-0.30$ we conclude that the scaling factor has a value $\frac{a+m_{\xi }-1}{2}\approx 0.62-0.65$, remarkably close to that observed in a number of works [11,15,17,19,21].

From Equation (9) we calculate the generalized Benioff strain rate dΩξdt:

(18) $$\frac{d\Omega_{\xi }\left(t\right)}{dt}=\gamma \frac{{\left(U_{0in}\right)}^{a}}{L_{0}^{a\left(d-d_{e}+1\right)}}{\left(\frac{t_{f}-t}{T_{c}}\right)}^{a\left(d_{e}-1-d\right)}$$

According to Equations (9) and (18) we have:

$$\Omega_{\xi }\left(t\right)=\Omega_{\xi f}-\;\gamma \frac{U_{0in}{}^{a}}{m_{\xi }}{\left(\frac{T_{c}}{L_{0}}\right)}^{1-m_{\xi }}{\left(t_{f}-t\right)}^{m_{\xi }}$$

and:

$$\frac{d\Omega_{\xi }\left(t\right)}{dt}=\gamma U_{0in}{}^{a}{\left(\frac{T_{c}}{L_{0}}\right)}^{1-m_{\xi }}\frac{1}{{\left(t_{f}-t\right)}^{1-m_{\xi }}}$$

When $t_{f}-t=T_{c}$ i.e., in the start of the accelerating deformation stage, we have:

(19) $$\Omega_{\xi l}=\Omega_{\xi f}-\gamma \frac{U_{0in}{}^{a}}{m_{\xi }}\frac{T_{c}}{L_{0}{}^{1-m_{\xi }}}$$

It is physically reasonable to expect a continuity of physical parameters in the transition from the linear to the accelerating deformation period and to accept that at $t=t_{f}-T_{c}$ a continuity of the generalized Benioff strain rate gives:

(20) $${\frac{d\Omega_{\xi }}{dt}|}_{l}={\frac{d\Omega_{\xi }}{dt}|}_{\left(t=\;t_{f}-T_{c}\right)}=\gamma U_{0in}{}^{a}\frac{1}{L_{0}{}^{1-m_{\xi }}}$$

where ${\frac{d\Omega_{\xi }}{dt}|}_{l}$ is the slope of the linear part. We calculate the mean generalized Benioff rate during the accelerated (deformed) period $T_{c}$ i.e., from $t=t_{f}-T_{c}$ to $t=t_{f}$:

(21) $${\langle \frac{d\Omega_{\xi }}{dt}\rangle }_{D}=\frac{1}{T_{c}}\overset{T_{c}}{\underset{t_{f}-T_{c}}{\int }}\frac{d\Omega_{\xi }\left(t\right)}{dt}dt=\gamma \frac{U_{0in}{}^{a}}{m_{\xi }}{\left(\frac{1}{L_{0}}\right)}^{1-m_{\xi }}$$

From (20) and (21) we reach the conclusion that: $\frac{{\langle \frac{d\Omega_{\xi }}{dt}\rangle }_{D}}{{\frac{d\Omega_{\xi }}{dt}|}_{l}}=\frac{1}{m_{\xi }}\approx 3-4$ Furthermore from (19), if $\Omega_{\xi l}\ll \Omega_{\xi f}$ we have: $\Omega_{\xi f}\approx \gamma \frac{U_{0in}{}^{a}}{m_{\xi }}\frac{T_{c}}{L_{0}{}^{1-m_{\xi }}}$. Combining the latter expression for $\Omega_{\xi f}$ with Equation (21) leads to:

(22) $$\Omega_{\xi f}\approx {\langle \frac{d\Omega_{\xi }}{dt}\rangle }_{D}T_{c}$$

which is exactly the expression proposed in [11,15,17,19,21]. Equation (22) could be written as:

$$\Omega_{\xi f}\approx \frac{1}{m_{\xi }}\left({\frac{d\Omega_{\xi }}{dt}|}_{l}\right)T_{c}$$

which indicates that if we estimate $T_{c}$ and the slope of the linear part of the generalized Benioff strain we can estimate at least the order of magnitude of $\Omega_{\xi f}$ at the time of failure.

Let us assume that the last earthquake prior the main shock appears at a time $t_{1}=t_{f}-\delta t_{1}$. Applying the time to failure Equation (9) we have:

$$\Omega_{\xi }\left(t_{1}\right)=\Omega_{\xi f}-\gamma \frac{U_{0in}{}^{a}}{m_{\xi }}{\left(\frac{T_{c}}{L_{0}}\right)}^{1-m_{\xi }}{\left(t_{f}-t_{1}\right)}^{m_{\xi }}$$

or:

$$\Omega_{\xi }\left(t_{1}\right)=\Omega_{\xi f}-\gamma \frac{U_{0in}{}^{a}}{m_{\xi }}{\left(\frac{T_{c}}{L_{0}}\right)}^{1-m_{\xi }}{\left(\delta t_{1}\right)}^{m_{\xi }}.$$

Even if our approach is general, from here on, we limit ourselves to the case *ξ* = 1/2 describing the Benioff strain which is very commonly applied. In this case the Benioff stain of the main shock is: $\Omega_{\frac{1}{2},m}=E_{m}{}^{1/2}=\Omega_{\frac{1}{2},f}-\Omega_{1/2}\left(t_{1}\right)$ resulting in:

(23) $$E_{m}{}^{1/2}=\gamma \frac{U_{0in}{}^{a}}{m_{1/2}}{\left(\frac{T_{c}}{L_{0}}\right)}^{1-m_{1/2}}{\left(\delta t_{1}\right)}^{m_{1/2}}={\langle \frac{d\Omega_{1/2}}{dt}\rangle }_{D}{\left(\frac{\delta t_{1}}{T_{c}}\right)}^{m_{1/2}}T_{c}=\frac{1}{m_{1/2}}\left({\frac{d\Omega_{1/2}}{dt}|}_{l}\right){\left(\frac{\delta t_{1}}{T_{c}}\right)}^{m_{1/2}}T_{c}$$

Since the seismic energy is related to seismic magnitude by the relation:

logE=1.5M+4.7

Equation (23) leads to:

(24) $$M_{m}=\frac{4}{3}\log\left[\frac{1}{m_{1/2}}\left({\frac{d\Omega_{1/2}}{dt}|}_{l}\right)T_{c}\right]+\frac{4m_{1/2}}{3}\log\left(\frac{\delta t_{1}}{T_{c}}\right)-3.13$$

Using previously published observational estimates [11,15,17,19,21] for *ξ* = 1/2 we have an order of magnitude estimation for the parameter ${\frac{d\Omega_{1/2}}{dt}|}_{l}\approx 10^{6}\;J^{\frac{1}{2}}/y$, and assuming $T_{c}\approx 5\;\mathrm{years}$ with $m_{1/2}=0.25-0.30$ and the last main preshock to appear at: $\frac{\delta t_{1}}{T_{c}}\approx 0.1$ (i.e., $\delta t_{1}\approx 0.5\;\mathrm{year}$), the expected earthquake magnitude of the main event should be of the order of $M_{m}\approx 6.3$. In addition, since $\frac{\delta t_{1}}{T_{c}}\leq 1$ from Equation (24) we have the constraint $M_{m}\leq \frac{4}{3}\log\left[\frac{1}{m_{1/2}}\left({\frac{d\Omega_{1/2}}{dt}|}_{l}\right)T_{c}\right]-3.13$, which leads to the conclusion that the maximum expected earthquake magnitude in an area with background Benioff strain rate ${\frac{d\Omega_{1/2}}{dt}|}_{l}$ is:

$$M_{m}^{max}=\frac{4}{3}\log\left[\frac{1}{m_{1/2}}\left({\frac{d\Omega_{1/2}}{dt}|}_{l}\right)T_{c}\right]-3.13$$

**Figure 7:**
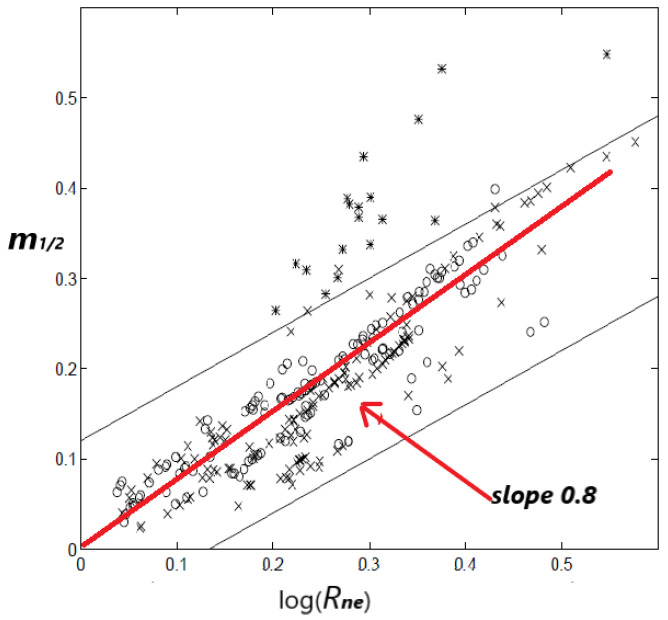
The *m*_1*/*2_ exponent vs. the logarithm of the normalized square root of energy released *R_ne_* as modified from [10]. The red line has slope close to 0.8 (see text).

It is obvious that the ratio $\frac{\delta t_{1}}{T_{c}}$ is a crucial parameter to define the final stage of the main event preparation. [10] proposed a relationship between $m_{1/2}$ and the normalized energy released *R_ne_* which is defined as the total cumulative square root energy (i.e., *ξ* = 1/2) divided by the square root of the energy released by the main shock. Thus $R_{ne}=\frac{\Omega \left(t_{f}\right)}{E_{m}^{1/2}}$. From the previous expressions it is obtained that $R_{ne}={\left(\frac{\delta t_{1}}{{Τ}_{c}}\right)}^{-m_{1/2}}$ which leads to:

$$m_{1/2}=\frac{1}{\log\left(\frac{{Τ}_{c}}{\delta t_{1}}\right)}\;logR_{ne}$$

in which a linear relation between *m*-parameter and $logR_{ne}$ is suggested with a positive slope $\frac{1}{\log\left(\frac{{Τ}_{c}}{\delta t_{1}}\right)}$ since *T_c_* > *δt_1_*). The Figure 7 from [10] is reproduced here (as Figure 7) and a red line with a slope of the order of 0.8–1.0 is added to the figure that seems to describe the majority of the data points. The latter slope permits an order of magnitude estimation of the ratio $\frac{\delta t_{1}}{{Τ}_{c}}$, leading to $\frac{\delta t_{1}}{{Τ}_{c}}=0.05-0.1$.

## 3. Concluding Remarks

The organization patterns that earthquakes and faults exhibit has motivated the statistical physics approach to earthquake occurrence [85]. Based on non-extensive statistical physics and the Tsallis entropy a framework that produces the collective pattern of seismicity has been introduced to describe the macroscopic behavior of complex seismic systems that present strong correlations among their elements [70]. Observational indications support the hypothesis that many large earthquakes are preceded by accelerating-decelerating seismic release rates which are described by a power law time to failure relation. The question whether accelerating/decelerating seismicity is described in terms of non-extensive statistical physics presents a challenge. This is the problem addressed in the present work. Motivated by a simple model originally proposed in [16] where fundamental concepts such as that of energy conservation are used to understand the accelerating seismicity we generalized it by introducing the concept of generalized Benioff strain which is merged with ideas of complexity and non-extensive statistical physics.

In the present work, a unified theoretical framework is discussed based on the ideas of non-extensive statistical physics along with fundamental principles of physics such as the energy conservation in a faulted crustal volume undergoing tectonic stress loading. We define a generalized Benioff strain function $\Omega_{\xi }\left(t\right)=\overset{n\left(t\right)}{\underset{i=1}{\sum }}E^{\xi }\left(t\right)$ where 0≤ξ≤1 and a time-to-failure power-law of $\Omega_{\xi }\left(t\right)$ derived using the fundamental principle of energy conservation in a fault system that obeys a hierarchical distribution law extracted from Tsallis entropy. In the time-to-failure power-law that $\Omega_{\xi }\left(t\right)$ follows the existence of a common exponent *m_ξ_* which is a function of the non-extensive entropic parameter *q* is demonstrated.

Since $\Omega_{\xi }\left(t\right)=\Omega_{\xi f}-B{\left(t_{f}-t\right)}^{m_{\xi }}$, with $\Omega_{\xi f}=\Omega \left(t=t_{f}\right),$ B = $\gamma \frac{T_{c}^{a\left(d-d_{e}+1\right)}{\left(U_{0in}\right)}^{a}}{L_{0}^{a\left(d-d_{e}+1\right)}}\frac{1}{a\left(d_{e}-1-d\right)+1}$ and:

$$m_{\xi }=a\left(d_{e}-1-d\right)+1=1-\alpha +\alpha \;d_{e}\frac{2-q}{q-1}=1+\alpha \;d_{e}\left(\beta -\frac{1}{d_{e}}\right)=1+\alpha \;\left(\frac{2}{3}b\;d_{e}-1\right)$$

the properties of these parameters have been studied and their upper and lower bound of the parameters *q* and *b* created according the geometrical limitations, the positive definition of *m_ξ_* and the condition of the system (accelerating with *m_ξ_* < 1 or decelerating with *m_ξ_* > 1). The range of *q* and *b* values that could drive the system into an accelerating stage and to failure has been discussed, along with the precursory variations of *m_ξ_* as resulting from the appearance of precursory *b*-value anomaly.

It has been proved that $\Omega_{\xi f}\approx {\langle \frac{d\Omega_{\xi }}{dt}\rangle }_{D}T_{c}$. where ${\langle \frac{d\Omega_{\xi }}{dt}\rangle }_{D}$ is the mean generalized Benioff sain rate during the accelerated (deformed) period $T_{c}$, while $\frac{{\langle \frac{d\Omega_{\xi }}{dt}\rangle }_{D}}{{\frac{d\Omega_{\xi }}{dt}|}_{l}}=\frac{1}{m_{\xi }}\approx 3-4$ where ${\frac{d\Omega_{\xi }}{dt}|}_{l}$ refers tthe generalized Benioff strain rate during the linear stationary part of $\Omega_{\xi }\left(t\right)$. A discussion of a number of empirical scaling laws is given, among them the scaling of *B* with the magnitude of the main even produced from first principles in agreement with the empirical one.

Our calculations based on Tsallis entropy and the energy conservation give a new view on the empirical laws presented in the literature, the associated average generalized Benioff strain rate during the accelerating period with the background rate and connected model parameters with the expected magnitude of the main shock. The need for accurate earthquake catalogues that will support (or not) further theoretical results which are still in debate [86], is stressed.

## References

[1] Sornette D. (2004). Critical Phenomena in Natural Sciences: Chaos, Fractals, Self organization and Disorder.

[2] Bak P., Tang C. (1988). Earthquakes as self organized critical phenomenon. J. Geophys. Res..

[3] Papadopoulos G.A. (1986). Long term earthquake prediction in western Hellenic arc. Earthq. Pred. Res..

[4] Bowman D.D., Quillon G., Sammis C.G., Sornette A., Sornette D. (1988). An observational test of the critical earthquake concept. J. Geophys. Res..

[5] Varnes D.G. (1989). Predicting Earthquakes by Analyzing Accelerating Precursory Seismic Activity. Pure Appl. Geophys..

[6] Bufe C.G., Varnes J.D. (1993). Predictive Modeling of the Seismic Cycle of the Greater San Francisco Bay Region. J. Geophys. Res..

[7] Mignan A. (2008). Non-Critical Precursory Accelerating Seismicity Theory (NC PAST) and limits of the power-law fit methodology. Tectonophysics.

[8] Mignan A. (2011). Retrospective on the Accelerating Seismic Release (ASR) hypothesis: Controversy and new horizons. Tectonophysics.

[9] Mignan A. (2012). Seismicity precursors to large earthquakes unified in a stress accumulation framework. Geophys. Res. Lett..

[10] Brehm D.J., Braile L.W. (1999). Refinement of the modified time-to-failure method for intermediate-term earthquake prediction. J. Seism..

[11] Papazachos B., Papazachos C. (2000). Accelerated preshock deformation of broad regions in the Aegean area. Pure Appl. Geophys..

[12] Rundle J.B., Klein W., Turcotte D.L., Malamud B.D. (2000). Precursory Seismic Activation and Critical-point Phenomena. Pure Appl. Geophys..

[13] Tzanis A., Vallianatos F., Makropoulos K. (2000). Seismic and electrical precursors to the 17-1-1983, M7 Kefallinia earthquake, Greece: Signatures of a SOC system. Phys. Chem. Earth Part A Solid Earth Geodesy.

[14] Bowman D.D., King G.C. (2001). Accelerating seismicity and stress accumulation before large earthquakes. Geophys. Res. Lett..

[15] Papazachos C., Papazachos B. (2001). Precursory accelerated Benioff strain in the Aegean area. Ann. Geophys..

[16] Di Giovambattista R., Tyupkin Y. (2001). An analysis of the process of acceleration of seismic energy emission in laboratory experiments on destruction of rocks and before strong earthquakes on Kamchatka and in Italy. Tectonophysics.

[17] Papazachos C.B., Karakaisis G.F., Savvaidis A., Papazachos B.C. (2002). Accelerating Seismic Crustal Deformation in the Southern Aegean Area. Bull. Seism. Soc. Am..

[18] Tzanis A., Vallianatos F. (2003). Distributed power-law seismicity changes and crustal deformation in the SW Hellenic Arc. Nat. Hazards Earth Syst. Sci..

[19] Scordilis E.M., Papazachos C.B., Karakaisis G.F., Karakostas V.G. (2004). Accelerating seismic crustal deformation before strong mainshocks in Adriatic and its importance for earthquake prediction. J. Seismol..

[20] Di Giovambattista R., Tyupkin Y. (2004). Seismicity patterns before the M = 5.8 2002, Palermo (Italy) earthquake: Seismic quiescence and accelerating seismicity. Tectonophysics.

[21] Papazachos C.B., Karakaisis G.F., Scordilis M.E., Papazachos B.C. (2005). Global observational properties of the critical earthquake model. Bull. Seism. Soc. Am..

[22] Mignan A., Bowman D., King G. (2006). An observational test of the origin of accelerating moment release before large earthquakes. J. Geophys. Res..

[23] Mignan A., King G.C., Bowman D. (2007). A mathematical formulation of accelerating moment release based on the stress accumulation model. J. Geophys. Res..

[24] Mignan A., Di Giovambattista R. (2008). Relationship between accelerating seismicity and quiescence, two precursors to large earthquakes. Geophys. Res. Lett..

[25] De Santis A., Cianchini G., Qamili E., Frepoli A. (2010). The 2009 L’Aquila (Central Italy) seismic sequence as a chaotic process. Tectonophysics.

[26] Sornette A., Sornette D. (1990). Discrete scale invariance, complex fractal dimensions, and log-periodic fluctuations in seismicity. Tectonophysics.

[27] Sornette D., Sammis C.G. (1995). Complex critical exponents from renormalization group theory of earthquakes: Implications for earthquake predictions. J. Phys. I.

[28] Jaume S.C., Sykes L.R. (1999). Evolving towards a critical point: A review of accelerating moment/energy release prior to large and great earthquakes. Pure Appl. Geophys..

[29] Vallianatos F., Benson P., Meredith P., Sammonds P. (2012). Experimental evidence of a non-extensive statistical physics behavior of fracture in triaxially deformed Etna basalt using acoustic emissions. Eurphys. Lett..

[30] Sornette D., Vanneste C. (1992). Dynamics and Memory Effects in Rupture of Thermal Fuse Networks. Phys. Rev. Lett..

[31] Varotsos P., Sarlis N.V., Skordas E.S., Uyeda S., Kamogawa M. (2011). Natural time analysis of critical phenomena. Proc. Natl Acad. Sci. USA.

[32] Ben-Zion Y., Lyakhovsky V. (2002). Accelerated Seismic Release and Related Aspects of Seismicity Patterns on Earthquake Faults. Pure Appl. Geophys..

[33] Turcotte D.L., Newman W.I., Gabrielov A. (2000). A Statistical Physics Approach to Earthquakes, Geocomplexity and the Physics of Earthquakes.

[34] Tsallis C. (1988). Possible generalization of Boltzmann-Gibbs statistics. J. Stat. Phys..

[35] Tsallis C. (2009). Introduction to Nonextensive Statistical Mechanics-Approaching a Complex World.

[36] Vallianatos F., Papadakis G., Michas G. (2016). Generalized statistical mechanics approaches to earthquakes and tectonics. Proc. R. Soc. A Math. Phys. Eng. Sci..

[37] Vallianatos F., Michas G., Papadakis G. (2015). A description of seismicity based on non-extensive statistical physics: A review. Earthquakes and Their Impact on Society.

[38] Sarlis N.V., Skordas E.S., Varotsos P.A. (2010). Nonextensivity and natural time: The case of seismicity. Phys. Rev. E.

[39] Sarlis N.V., Skordas E.S., Varotsos P.A., Nagao T., Kamogawa M., Uyeda S. (2015). Spatiotemporal variations of seismicity before major earthquakes in the Japanese area and their relation with the epicentral locations. Proc. Natil Acad. Sci. USA.

[40] Papadakis G., Vallianatos F., Sammonds P. (2014). A Nonextensive Statistical Physics Analysis of the 1995 Kobe, Japan Earthquake. Pure Appl. Geophys..

[41] Papadakis G., Vallianatos F., Sammonds P. (2016). Non-extensive statistical physics applied to heat flow and the earthquake frequency-magnitude distribution in Greece. Phys. A Stat. Mech. Appl..

[42] Vallianatos F., Sammonds P. (2013). Evidence of non-extensive statistical physics of the lithospheric instability approaching the 2004 Sumatran-Andaman and 2011 Honshu mega-earthquakes. Tectonophysics.

[43] Telesca L. (2012). Maximum likelihood estimation of the nonextensive parameters of the earthquake cumulative magnitude distribution. Bull. Seismol. Soc. Am..

[44] Sotolongo-Costa O., Posadas A. (2004). Fragment-Asperity Interaction Model for Earthquakes. Phys. Rev. Lett..

[45] Silva R., Franca G.S., Vilar C.S., Alcaniz J.S. (2006). Nonextensive models for earthquakes. Phys. Rev. E.

[46] Chochlaki K., Vallianatos F., Michas G. (2018). Global regionalized seismicity in view of Non-Extensive Statistical Physics. Phys. A Stat. Mech. Appl..

[47] Telesca L. (2010). Nonextensive analysis of seismic sequences. Phys. A Stat. Mech. Appl..

[48] Vallianatos F. (2009). A non-extensive approach to risk assessment. Nat. Hazards Earth Syst. Sci..

[49] Vallianatos F. (2013). On the statistical physics of rockfalls: A non-extensive view. Europhys. Lett..

[50] Vallianatos F., Sammonds P. (2010). Is plate tectonics a case of non-extensive thermodynamics?. Phys. A Stat. Mech. Appl..

[51] Vallianatos F. (2011). A non-extensive statistical physics approach to the polarity reversals of the geomagnetic field. Phys. A Stat. Mech. Appl..

[52] Vallianatos F., Triantis D., Sammonds P. (2011). Non-extensivity of the isothermal depolarization relaxation currents in uniaxial compressed rocks. Europhys. Lett..

[53] Yamasaki K., Nanjo K.Z. (2009). A new mathematical tool for analyzing the fracture process in rock: Partial symmetropy of microfracturing. Phys. Earth Planet. Int..

[54] Vallianatos F. (2017). Transient Electromagnetic Method in the Keritis basin (Crete, Greece): Evidence of hierarchy in a complex geological structure in view of Tsallis distribution. Ann. Geophys..

[55] Vallianatos F., Sammonds P. (2011). A non-extensive statistics of the fault-population at the Valles Marineris extensional province, Mars. Tectonophysics.

[56] Vallianatos F., Kokinou E., Sammonds P. (2011). Non Extensive statistical physics approach to fault population distribution. A case study from the Southern Hellenic Arc (Central Crete). Acta Geophys..

[57] Michas G., Vallianatos F., Sammonds P. (2015). Statistical Mechanics and scaling of fault population with increasing strain in the Corinth Rift. Earth Planet. Sci. Lett..

[58] Nuannin P., Kulhánek O., Persson L. (2005). Spatial and temporal *b* value anomalies preceding the devastating off coast of NW Sumatra earthquake of 26 December 2004. Geophys. Res. Lett..

[59] Scordilis E.M. (2006). Decelerating precursory seismicity in Vrancea. Tectonophysics.

[60] Nuannin P., Kulhánek O., Persson L. (2012). Variations of *b*-values preceding large earthquakes in the Andaman-Sumatra subduction zone. J. Asian Earth Sci..

[61] Papadopoulos G.A., Minadakis G. (2016). Foreshock Patterns Preceding Great Earthquakes in the Subduction Zone of Chile. Pure Appl. Geophys..

[62] Kulhanek O., Persson L., Nuannin P. (2018). Variations of *b*-values preceding large earthquakes in the shallow subduction zones of Cocos and Nazca plates. J. South Am. Earth Sci..

[63] Scholz C.H., Aviles C.A., Das S., Boatwright J., Scholz C.H. (2013). The Fractal Geometry of Faults and Faulting. Earthquake Source Mechanics AGU Geophysical Monograph Series.

[64] Voight B. (1989). A relation to describe rate-dependent material failure. Science.

[65] Shcherbakov R., Turcotte D.L. (2003). Damage and Self-similarity in fracture. Theor. Appl. Fract. Mech..

[66] Turcotte D.L., Newman W., Shcherbakov R. (2003). Micro and macroscopic models of rock fracture. Geophys. J. Int..

[67] Nanjo K.Z. (2017). A fiber-bundle model for the continuum deformation of brittle material. Int. J. Fract..

[68] Brouers F., Sotolongo-Costa O. (2005). Relaxation in heterogeneous systems: A rare events dominated phenomenon. Phys. A Stat. Mech. Appl..

[69] Vallianatos F., Baziotis I., Udry A., Taylor L.A. (2014). Application of non-extensive statistical physics on Martian nakhlites: A first-order approach on the crystal size distribution of pyroxene using Tsallis entropy. Europhys. Lett..

[70] Vallianatos F., Michas G., Papadakis G., Chelidze T., Vallianatos F., Telesca L. (2018). Non Extensive Statistical Seismology: An Overview in Complexity of Seismic Time Series Measurement and Application.

[71] Saltas V., Vallianatos F., Triantis D., Stavrakas I., Chelidze T., Vallianatos F., Telesca L. (2018). Complexity in Laboratory Seismology: From Electrical and Acoustic Emissions to fracture, in Complexity of Seismic Time Series. Measurement and Application.

[72] Tyupkin Y.S. (2013). Manifestation of Self—Similar Structure in Foreshock and Aftershock Seismicity. Comput. Seism. Geodyn..

[73] Sornette D. (1998). Discrete scale invariance and complex dimensions. Phys. Rep..

[74] Sornette A., Sornette D. (1989). Self-organized criticality and earthquakes. Eur. Lett..

[75] Kagan Y.Y. (2010). Earthquake size distribution: Power-law with exponent *β* ≡ 1/2?. Tectonophysics.

[76] Kanamori H. (1978). Quantification of earthquakes. Nature.

[77] Papadopoulos G.A. (1988). Long-term accelerating foreshock activity may indicate the occurrence time of a strong shock in the Western Hellenic Arc. Tectonophysics.

[78] Papadopoulos G.A., Charalampakis M., Fokaefs A., Minadakis G. (2010). Strong foreshock signal preceding the L’Aquila (Italy) earthquake (M_w_6.3) of 6 April 2009. Nat. Hazards Earth Syst. Sci..

[79] Papadopoulos G.A., Drakatos G., Plessa A. (2000). Foreshock activity as a precursor of strong earthquakes in Corinthos Gulf, Central Greece. Phys. Chem. Earth Part A Solid Earth Geodesy.

[80] Wang J.H. (2016). A mechanism causing *b*-value anomalies prior to a mainshock. Bull. Seism. Soc. Am..

[81] Schorlemmer D., Wiemer S. (2005). Microseismicity data forecast rapture area. Nature.

[82] Nanjo K.Z., Hirata N., Obara K., Kasahara K. (2012). Decade-scale decrease in *b* value prior to the *M9*-class 2011 Tohoku and 2004 Sumatra quakes. Geophys. Res. Lett..

[83] Lei X. (2003). How do asperities fracture? An experimental study of unbroken asperities. Earth Planet. Sci. Lett..

[84] Xue Y., Liu J., Yu H., Liu S.Q. (2012). Seismicity characteristics of the 2011 M9.0 Tohuku earthquake near the East Cost of Honshu in Japan. Chin. Sci. Bull..

[85] Sornette D., Werner M.J., Meyers R.A. (2009). Statistical physics approaches to seismicity. Encyclopedia of Complexity and Systems Science.

[86] Mignan A. (2014). The debate on the prognostic value of earthquake foreshocks: A meta-analysis. Sci. Rep..

